# Art and Disease

**Published:** 1894-03-17

**Authors:** 


### Art and Disease.

### VIII.—IN THE NATIONAL GALLERY.

It has been seen that painters and sculptors, from the very earliest times, have shown a quick eye for noting the effects on the human frame of any deficiency, whether mental or bodily, and have not shrunk from admitting into their works the ravages of disease and death itself. It is interesting to note what examples of this are to be found in the national collection.

The earliest work presenting points of interest in this connection is one by Duccio di Buoninengna, who flourished towards the close of the thirteenth century. He represents Christ healing the blind, and seems to have been curiously divided between convention and reality in his conception of the subject. The miracle takes place in the courtyard of a monastery, and the background consists of a very delicate bit of early Italian architecture. Christ and the disciples are in a kind of Eastern dress, and their feet are bare. The two young men on whom the miracle is being worked are, however, in the familiar Italian dress of the period, consisting of a three-quarter tunic with a girdle, and with a cloak thrown over the shoulders. They are dressed in crimson and grey, exactly alike in all points, suggesting the existence of some liveried order of licensed beggars. Each has high red buskins reaching nearly to the knee, and a wallet hanging on the left side. They are provided with long poles, curiously forked at the top. One man who has received his sight has thrown down his pole, and has turned aside to gaze with upraised hands in a kind of rapture at the sky. The other is receiving the healing touch from the hand of Christ, and is standing motionless, holding firmly his accustomed prop.

A remarkable picture of the German school, in the fifteenth century, representing the death of the Virgin, seems to take the spectator into the very midst of a real sick room. The dying woman is suffering acutely, her white face is drawn into an unmistakeable look of anguish, and her hands are tightly clenched as though to repress a cry. Overhead in a vision, but not perceived by her, the Saviour with attendant angels is waiting to receive her spirit. A tonsured priest is approaching a holy taper, and other priests are kneeling in prayer round the bed; one by a quaint anachronism is occupied in telling his beads. The physician in a long white robe, from which depend two pomanders of silver essence cases, is preparing with grave concern some arrangement for relieving his patient in a bucket held by an assistant. In the background another assistant with inflated cheeks is kindling a chafing dish on which probably some reviving preparation has been sprinkled. The effect of it all is extraordinarily life-like and dramatic, and with all the abundance of detail the interest is entirely centred on the death-pangs of the saint, while the heavenly vision above lends hope and spirituality to the motive of the picture.

Several examples of the painful realism alluded to in a former article as exhibited by some early artists in studies of the Crucifixion will be found in the gallery; no one will care to linger long over them. One may be mentioned ("The Dead Christ." Lombard School, sixteenth century) from the very remarkable effect conveyed of the amputation of the arm above the elbow, which can obviously not have been intended by the artist. A deposition by Lambert Lombard, dating from about the middle of the sixtenth century, is a notable instance of the extreme emaciation which appears to have been studied *con amore* by mediæval painters.

The exhumation of St. Hubert, by a Flemish artist of the fifteenth century, is a scene full of animation, and the face and hands of the dead saint, whose body is reposing in the newly uncovered coffin before the altar, are drawn with such minute care and resemblance to the appearance of an embalmed body as to suggest that the artist had had the opportunity of drawing from personal observation. On the other hand, in the large picture of "San Zenobia restoring a dead child to life," it is clear that the artist was at no pains to place his model in an attitude even possible, not to say natural, for a lifeless body. The boy is half-lying on a crimson cushion before the saint, whose hands are upraised in prayer. The mother is kneeling, too, with her eyes on the saint. The miracle has not yet taken place, for one of the assistants is gazing with grave pity on the child and shows no sign of the awe which symptoms of returning life would occasion. Yet the boy, in contradiction to the death pallor of his face, could only maintain his position by actual muscular exertion.

A martyrdom of Saint Sebastian, by Antonio Pollaiuolo, forcibly illustrates Ruskin's criticism of this favourite subject. The absurdity of the picture as a representation of a violent death, consists not only in the evident unconcern of the saint about the arrows which have lodged in his body, but in the fact that the arrows are being shot at so close a range as almost to graze his feet before the bow is drawn. A beautiful still landscape, of Giovanni Bellini, where in the foreground a kneeling saint is receiving with wrapt devotion the death thrust of a robber, displays this suppression of any token of violence in its highest aspect.

The celebrated "Plague at Ashdod" should be studied as a typical example of the incidents common to most plague pictures. The accuracy of observation which Rubens reveals in all his studies of infirmities is amply illustrated in his "Conversion of Saint Bavon," where hysteria, lameness, ricketts, and spinal curvature all find a place among the crowd waiting to be healed, and are delineated with loving exactitude.

An imposing medical professor of the German School, 16th century, must not be passed over, though connected only indirectly with our subject. The magnificent black robe, lined with costly fur, the massive rings on the first and third fingers of either hand, the stately ruff, and still more the lofty dignity of his whole air, bespeak him a member of a wealthy and important profession. The right hand rests lightly on a skull.

